# Isolation and Characterization of a Metastatic Hybrid Cell Line Generated by ER Negative and ER Positive Breast Cancer Cells in Mouse Bone Marrow

**DOI:** 10.1371/journal.pone.0020473

**Published:** 2011-06-01

**Authors:** Keya De Mukhopadhyay, Abhik Bandyopadhyay, Ting-Tung A. Chang, Abdel G. Elkahloun, John E. Cornell, Junhua Yang, Beth A. Goins, I-Tien Yeh, Lu-Zhe Sun

**Affiliations:** 1 Department of Cellular and Structural Biology, University of Texas Health Science Center, San Antonio, Texas, United States of America; 2 Department of Radiology, University of Texas Health Science Center, San Antonio, Texas, United States of America; 3 Genome Technology Branch, National Human Genome Research Institute, National Institutes of Health, Bethesda, Maryland, United States of America; 4 Department of Epidemiology and Biostatistics, University of Texas Health Science Center, San Antonio, Texas, United States of America; 5 Department of Pathology, University of Texas Health Science Center, San Antonio, Texas, United States of America; Virginia Commonwealth University, United States of America

## Abstract

**Background:**

The origin and the contribution of breast tumor heterogeneity to its progression are not clear. We investigated the effect of a growing orthotopic tumor formed by an aggressive estrogen receptor (ER)-negative breast cancer cell line on the metastatic potential of a less aggressive ER-positive breast cancer cell line for the elucidation of how the presence of heterogeneous cancer cells might affect each other's metastatic behavior.

**Methods:**

ER positive ZR-75-1/GFP/puro cells, resistant to puromycin and non-tumorigenic/non-metastatic without exogenous estrogen supplementation, were injected intracardiacally into mice bearing growing orthotopic tumors, formed by ER negative MDA-MB-231/GFP/Neo cells resistant to G418. A variant cell line B6, containing both estrogen-dependent and -independent cells, were isolated from GFP expressing cells in the bone marrow and re-inoculated in nude mice to generate an estrogen-independent cell line B6TC.

**Results:**

The presence of ER negative orthotopic tumors resulted in bone metastasis of ZR-75-1 without estrogen supplementation. The newly established B6TC cell line was tumorigenic without estrogen supplementation and resistant to both puromycin and G418 suggesting its origin from the fusion of MDA-MB-231/GFP/Neo and ZR-75-1/GFP/puro in the mouse bone marrow. Compared to parental cells, B6TC cells were more metastatic to lung and bone after intracardiac inoculation. More significantly, B6TC mice also developed brain metastasis, which was not observed in the MDA-MB-231/GFP/Neo cell-inoculated mice. Low expression of ERα and CD24, and high expression of EMT-related markers such as Vimentin, CXCR4, and Integrin-β1 along with high CD44 and ALDH expression indicated stem cell-like characteristics of B6TC. Gene microarray analysis demonstrated a significantly different gene expression profile of B6TC in comparison to those of parental cell lines.

**Conclusions:**

Spontaneous generation of the novel hybrid cell line B6TC, in a metastatic site with stem cell-like properties and propensity to metastasize to brain, suggest that cell fusion can contribute to tumor heterogeneity.

## Introduction

Breast cancer is the most frequent malignant disease in women, affecting 1 in 8 North American women throughout their lifetime and is the second leading cause of cancer-related deaths in U.S. [1]. Mechanisms for the frequent failure of chemotherapy, endocrine therapy or immunotherapy to successfully treat breast cancer are elusive and under active investigation.

Breast cancer cells in a patient are heterogeneous, differing in their apparent state of differentiation and malignant potential. Random mutational events and/or epigenetic changes of cancer cells followed by the selection of more malignant variants or acquisition of stem cell like properties has been believed to be the mechanism for tumor progression and consequently for the generation of heterogeneous tumor cell population. An alternative cell fusion model of cancer progression and metastasis has also been proposed rather than progressive accumulation of genetic or epigenetic alterations in a single cell lineage [2], [3]. Rapid acquisition of metastatic phenotypes has recently been shown through fusion between tumor cells [4] or between tumor cells and bone marrow derived cells [5] indicating a potentially important role of cell fusion in the progression and phenotypic diversity of cancer.

One potential contribution of the heterogeneity to tumor progression is the production of various secreted factors from different types of tumor cells, which may promote malignant behavior among themselves. An emerging paradigm is that tumors are able to produce factors that induce the formation of so-called pre-metastatic niches in organs where metastases will ultimately develop [6]. The present study was initiated to determine whether a growing orthotopic tumor formed by an aggressive ER-negative breast cancer cell line might affect the metastatic potential of a less aggressive ER-positive breast cancer cell line. To test this hypothesis, five-week-old female nude mice were injected orthotopically with highly aggressive ER-negative human breast cancer MDA-MB-231/GFP/Neo cells. After three weeks, less aggressive human breast cancer ER-positive ZR-75-1/GFP/puro cells were inoculated into these tumor bearing mice via intra-cardiac (IC) route. Puromycin resistant metastatic ZR-75-1/GFP/puro cells were obtained from the bone marrow of one mouse and established as a variant cell line called B6. B6 cells were found to be a heterogeneous population containing both estrogen-dependent and -independent cells when tested for their tumorigenicity in nude mice with or without estrogen supplementation. We show that the estrogen-independent cells, isolated from an estrogen-independent tumor, is a novel hybrid cell line generated spontaneously in a metastatic site (mouse bone marrow), which has propensity to metastasize to brain in addition to lung and bone. The cell line, named B6TC, exhibits phenotypes of CD44^hi^CD24^lo^, high expression of ALDH, and formation of mammospheres, all of which have been shown to be properties of breast cancer stem cells. Our results indicate that the secreted factors from the highly aggressive growing tumors might have induced an appropriate environment for the less aggressive tumor cells to metastasize in the bone resulting in the formation of the hybrid cell line, which could cause additional tumor heterogeneity.

## Results

### Generation of bone metastasis from less aggressive breast cancer cells in the presence of highly aggressive, orthotopic tumors

**Figure 1:**
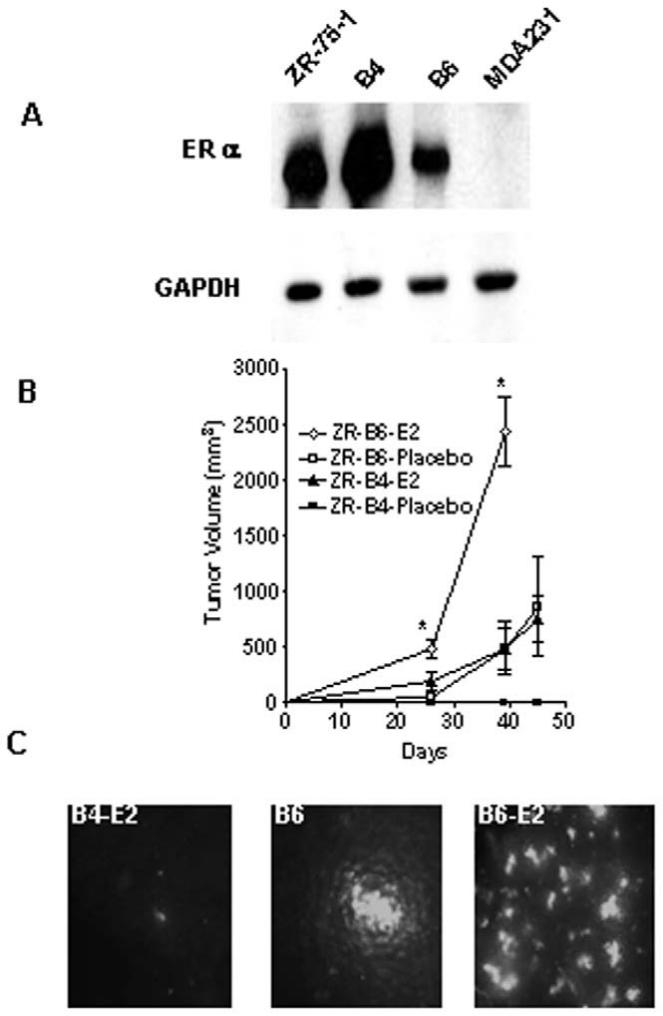
Tumorigenicity and metastatic potential of B4 and B6 cells. (A) Western blot analysis of ERα in the cell extracts of ZR-75-1, B4, B6, and MDA-MB-231 cells. Glyceraldehyde-3-phosphate dehydrogenase (GAPDH) levels were also measured in cell lysates to show equal sample loading. (B) B4 and B6 cells were separately inoculated into the both sides of inguinal mammary fat pad (2×10^6^ cells) of 5-week-old female athymic nude mice in presence of 17β-estradiol (E2) pellets (0.72 mg/90 days) or placebo pellets. The tumor sizes were measured with a caliper and tumor volumes were calculated with the equation V  =  (L×W^2^) x 0.5, where L is length and W is width of a tumor. The asterisk “*” indicates significant difference at P<0.05 between the mean tumor volumes of B6-E2 and B6-placebo at the given time points. For the B4 cell-inoculated mice, only the E2 group showed tumor, but not the placebo group. (C) Representative pictures of lung metastasis of the B4-E2, B6-Placebo and B6-E2 group as detected by GFP fluorescence.

Experimental evidence indicates that soluble factors produced by aggressive cancer cells may generate metastatic niches for less aggressive cancer cells, and we were interested to determine if growing orthotopic tumor formed by an aggressive ER-negative breast cancer cell line could affect the metastatic potential of a less aggressive ER-positive breast cancer cell line. Five-week-old female athymic nude mice (five in each group) were inoculated orthotopically into the mammary fat pad with ER-negative and metastatic MDA-MB-231/GFP/Neo or MDA-MB-435-F-L/GFP cells. ER-positive human breast cancer ZR-75-1/GFP/puro cells, which we know are non-tumorigenic and non-metastatic without exogenous estrogen supplementation in nude mice, were then injected intracardiacally into mice bearing growing tumors formed by MDA-MB-231 or MDA-MB-435-F-L cells two and half weeks after the orthotopic inoculation. We found ZR-75-1/GFP/puro cells in bone marrow of two mice bearing growing MDA-MB-231/GFP/Neo orthotopic tumors and one mouse bearing growing MDA-MB-435-F-L/GFP orthotopic tumors in the absence of estrogen supplementation, but not in control mice without orthotopic tumors. The metastatic ZR-75-1/GFP/puro cells were flushed out of the bone marrows and established as variant cell lines. Two variant lines, called B4 and B6, from two mice bearing MDA-MB-435-F-L/GFP or MDA-MB-231/GFP/Neo tumors respectively were further characterized for their ER expression, tumorigenicity in the mouse mammary fat pad, and metastatic potential to lung and bone through intracardiac injection in the presence or absence of estrogen supplementation. B4 cells expressed more ERα in Western immunoblot analysis compared to B6 (Fig. 1A). Surprisingly, although both variants were isolated from bone marrow and they showed the same anchorage-dependent and -independent growth property in vitro (data not shown), B6 was significantly more tumorigenic than B4 both with or without estrogen supplementation in vivo (Fig. 1B). In fact, B6 was more or equally tumorigenic and metastatic to lung (Fig. 1C) in the absence of estrogen supplementation as B4 in the presence of estrogen supplementation. Like parental ZR-75-1 cell, B4 is not tumorigenic and metastatic without estrogen supplementation (Fig. 1B).

### Isolation and characterization of an estrogen-independent ZR-75-1 cell line

The B6 cells were found to contain both estrogen-dependent and –independent cells when they were orthotopically injected into female nude mice with or without estrogen supplementation (Fig. 1B). We isolated and established a cell line from a tumor with estrogen supplementation and called it B6TE and another cell line from a tumor without estrogen supplementation and called it B6TC. More interestingly, both B6 and B6TC cells were found to contain cells resistant to both puromycin and G418. The majority of B6TC cells were resistant to both antibiotics whereas a small percent of B6 cells were resistant to both antibiotics suggesting that the double resistant cells were likely derived from a fusion cell containing genetic materials of both MDA-MB-231/GFP/Neo and ZR-75-1/GFP/puro cells. We mixed MDA-MB-231/GFP/Neo and ZR-75-1/GFP/puro cells in culture for more than one month and failed to obtain cells with resistance to both puromycin and G418 and therefore concluded that the fusion took place in the mouse bone marrow. To further strengthen our inference about the chimeric nature of the B6TC cell line, we performed gene microarray analysis of all three cell lines and generated a heat map (Fig. S1A) to show the level of expression of different genes across these three cell lines. The results from triplicate microarray chips revealed that B6TC cell has a different gene expression profile from those of its two parent cell lines even though it appears more closely related to the MDA-MB-231 cell than to the ZR-75-1 cell. Detailed analysis of gene expression levels revealed that significant differences in gene expression at the false discovery rate (FDR) of equal or smaller than 0.05 among all three cell lines was detected by 7,491 gene probes (Fig. S1B). In addition, significantly different levels of gene expression for B6TC were detected by 483 probes, for MDA-MB-231 were detected by 445 probes, and for ZR-75-1 were detected by 2,624 probes (Fig. S1B). Consistent with our conclusion that B6TC cell is derived from MDA-MB-231 cell, both cell lines contain a mutant p53^R280K^ (Fig. S2), which is known to be associated with MDA-MB-231 cell [7].

**Figure 2:**
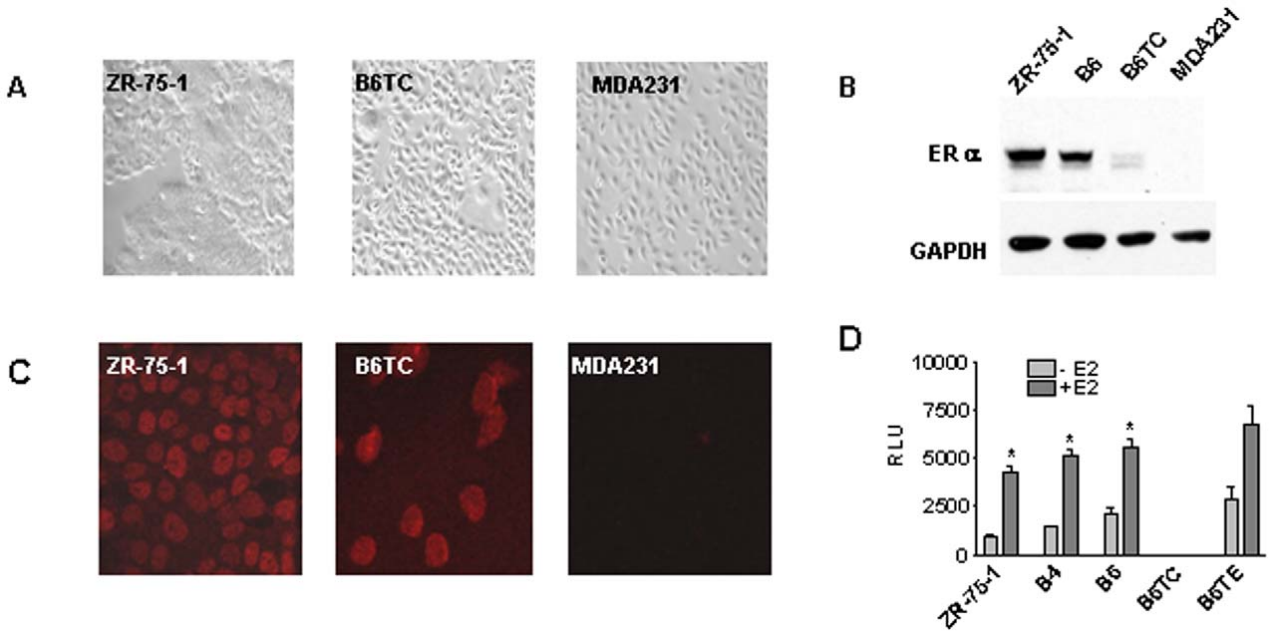
Characterization of estrogen-independent B6TC cells. (A) Morphology of ZR-75-1, B6TC and MDA-MB-231 cells as detected under bright field. (B) Western blot analysis in the cell extracts of the parental ZR-75-1, B6, B6TC and MDA-MB-231 control cells with antibodies to ER-α. GAPDH levels were measured in cell lysates to show equal sample loading. (C) Representative immunocytochemical staining pictures for the expression of ERα in ZR-75-1, B6TC and MDA-MB-231 cells were taken in confocal microscope under 60X magnification. (D) To confirm the expression of a functional ERα, the cells were transiently cotransfected with an estrogen-responsive promoter-luciferase report plasmid (ERE-thymidine kinase-Luciferase) and a β-gal expression plasmid. Cells were treated with 10^-7^ M E2. Luciferase and β-gal activities in cell lysates were determined. Luciferase activity normalized with β-gal activity is presented as the mean±SEM for each treatment from triplicate transfections. The asterisk “*” indicates significant difference between the –E2 and +E2 groups with Student *t* tests at P<0.05.

Morphologically the B6TC cells appear spindle-like, similar to MDA-MB-231, and differ from their parental ZR-75-1 cells, which are cobblestone-like (Fig. 2A). However, unlike MDA-MB-231 cells, which do not express ERα, B6TC cells express a low level of ERα, which is highly expressed in ZR-75-1 and B6 cells (Fig. 2B). Immunocytochemical staining of ERα revealed that while parental ZR-75-1 cells expressed a uniformly high level of ERα and MDA-MB-231 cells did not express ERα, B6TC cells are heterogeneous in ERα expression with the majority of cells expressing little or no ERα (Fig. 2C). When transfected with an estrogen-responsive promoter-luciferase reporter construct, luciferase activity was not detectable in B6TC cells treated with or without estradiol, whereas other ERα positive cell lines showed high levels of luciferase activity, which was further stimulated after treatment with estrogen (Fig. 2D) suggesting that the ERα-expressing cells of the B6TC line are too few to generate a detectable response.

### Growth properties of B6TC cells in vitro and in vivo

**Figure 3:**
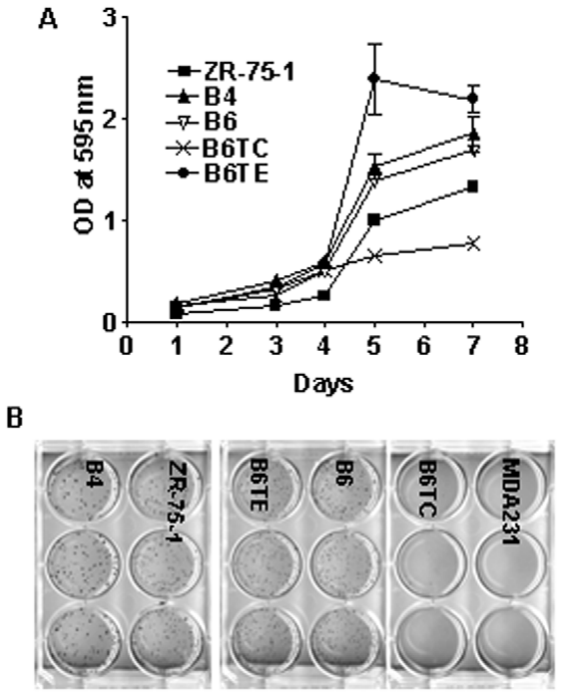
Comparison of in vitro growth property of B6TC cell with other cells. (A) Cells were plated in a 96-well plate at 2,000 cells per well. After 5 days of incubation, relative cell number in each well was determined with MTT assay as reflected with absorbance at 595 nm. The data are presented as mean±SEM from four replicate wells. (B) Anchorage independent growth property of the depicted cell lines was compared with a soft agarose assay. The cells were suspended in 0.4% soft agarose and plated at 1,000 cells per well. After 3 weeks of incubation, the colonies were visualized with *p*-iodonitrotetrazolium violet staining.

**Figure 4:**
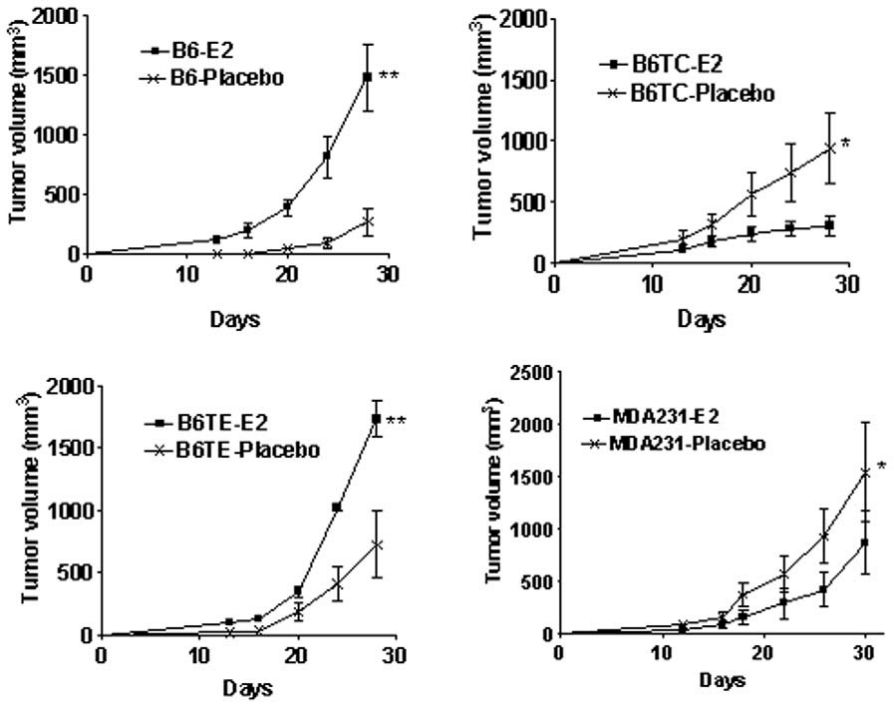
Comparison of in vivo tumorigenic property of B6TC cell with other cells. Exponentially growing tumor cells (2×10^6^) were inoculated into both inguinal mammary fat pads of 5-week-old female athymic nude mice implanted subcutaneously with a 17β-estradiol (E2) pellet (0.72 mg/90 days) or placebo pellet. The tumor sizes were measured with a caliper in two dimensions once every week after the growth of tumors was observed. Tumor volume was the sum of two tumors in each mouse and calculated with the equation V  =  (L×W^2^) x 0.5, where L is length and W is width of a tumor. Values are mean ± SEM of 5 mice in each group except the B6TE-E2 group, which had 4 mice. The asterisk indicates significant difference between the growth curves with E2 or placebo pellets by a paired Student *t* test at P<0.05 (*) or P<0.1 (**).

The B6TC cells grew slower on plastic compared to B6 and B6TE cells (Fig. 3A). When suspended in soft agarose, B6TC cells also formed smaller and fewer colonies than ZR-75-1, B4, B6, and B6TE cells, similar to MDA-MB-231 cells (Fig. 3B). When inoculated orthotopically in the mammary fat pad area, the tumors formed by B6TC cells grew at a similar rate as B6TE tumors, but faster than B6 tumors and slower than MDA-MB-231 tumors in the absence of exogenous estrogen supplementation (Fig. 4). However, in the presence of supplemented estrogen, the growth of B6TC tumors was significantly inhibited, similar to that of MDA-MB-231 tumors, whereas the growth of B6 and B6TE tumors was stimulated suggesting that B6TC cells behave more like ER negative breast cancer cells than ER positive breast cancer cells.

### B6TC cell induced more extensive bone metastases than MDA-MB-231 cell

**Figure 5:**
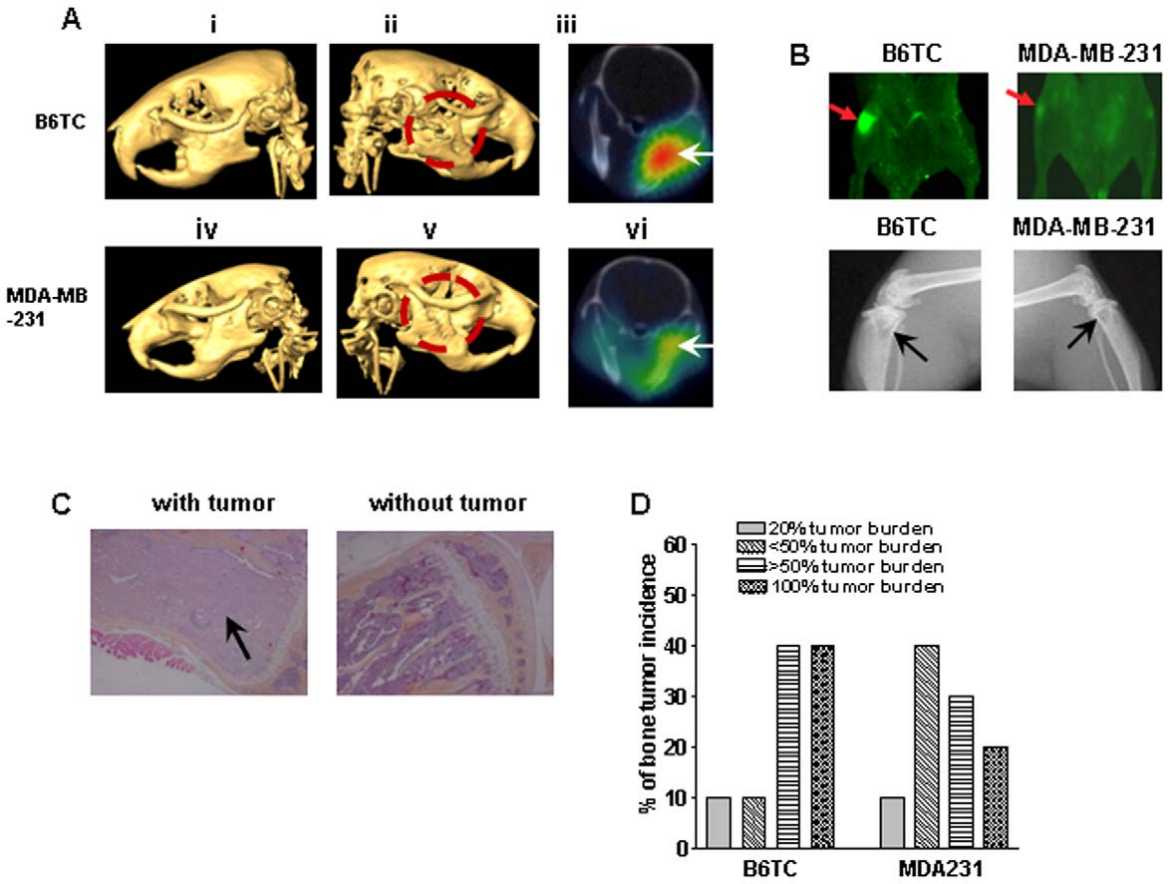
Comparison of bone metastatic potential between B6TC and MDA-MB-231 cells. B6TC and MDA-MB-231 cells were inoculated through the left cardiac ventricle of female nude mice at 0.1×10^6^ cells/mouse. Osteolytic bone metastases induced by B6TC and MDA-MB-231 cells were detected with various imaging methods. (A) Representative pictures of bone metastasis of the B6TC and MDA-MB-231 cells as detected by microCT imaging. Panel ii shows an osteolytic metastasis in the right jaw of a B6TC mouse while the left jaw (Panel i) remained intact. Similarly Panel v shows an osteolytic metastasis in the right jaw of a MDA-MB-231 mouse while the left jaw (Panel iv) remained intact. Axial PET/CT fused images are shown in Panels iii and vi for the B6TC and MDA-MB-231 mice, respectively. (B) Metastases in tibia/femur were detected by whole mouse GFP imaging as shown with a representative image for a B6TC mouse and a MDA-MB-231 mouse in the upper panels. Bone resorption due to osteolysis in the tibia was detected with X-ray radiographs for the B6TC and MDA-MB-231 mice as shown in the lower panels respectively. (C) At the termination of the experiment, the femora and tibiae were fixed in buffered formalin and decalcified. Paraffin-embedded sections were stained with hematoxylin, eosin, orange G, and phloxine. Representative pictures of the stained tibial sections with tumor indicated by arrow and without tumor are shown in the two panels respectively. (D) Mouse tibiae with tumor were divided into four groups and the results were expressed in percentage of tibiae with <20% tibia replaced by tumor, with 20–50% tibia replaced by tumor, with >50% of tibia replaced by tumor, and with 100% of tibia replaced by tumor.

Since B6TC cell was derived from MDA-MB-231 cell, which is known to generate osteolytic bone metastasis, we used an intracardiac injection model of experimental metastasis to compare their ability to induce bone metastasis. Female nude mice were inoculated with MDA-MB-231 and B6TC cells through the left ventricle of the heart. Bone metastases induced by both cell lines were monitored by whole-mouse GFP imaging, as described earlier [8], and PET and CT imaging. Bone metastases in the mandibles by the B6TC and MDA-MB-231 cells as detected by CT imaging (Fig. 5A i, ii, iv, and v) and PET imaging (Fig. 5A iii and vi) revealed more extensive osteolytic metastases by B6TC cells (Fig. 5A ii and iii) than by MDA-MB-231 cells (Fig. 5A v and vi). Figures 5Ai and 5Aiv show the intact mandible bones without metastases on the left side of the respective mouse inoculated with either MDA-MB-231 cells or B6TC cells. Whole-mouse GFP imaging revealed more extensive tibial metastases indicated by the presence of GFP positive tumors in the B6TC group as shown with a representative image in Fig. 5B in comparison to the MDA-MB-231 group. Both B6TC and MDA-MB-231 cells also induced osteolysis in the tibiae as detected by Faxitron X-ray imaging (Fig. 5B). We also examined the tumor burden in stained histologic sections of the femora and tibiae of mice inoculated with B6TC or MDA231-MB-231 cells. Representative bone histology pictures with or without metastatic tumor are shown in Fig. 5C. Comparison of the percent incidence of different levels of tibial tumor burden showed that 40% of the tibiae from the B6TC mice had greater than 50% of its marrow replaced by tumor and another 40% tibia was 100% filled with tumor in their marrow whereas the incidences for the tibia from MDA-MB-231 mice for 50% and 100% filled with tumors were only 30% and 20% respectively (Fig. 5D). In the B6TC group due to the extensive metastases of tumor cells in the leg bones, seven mice became paralyzed in the hind limbs compared to the three mice with similar condition in the MDA-MB-231 group. These observations suggest that B6TC cell has a higher potential in inducing osteolytic bone metastases than MDA-MB-231 cell.

### B6TC cell induced brain metastases

**Figure 6:**
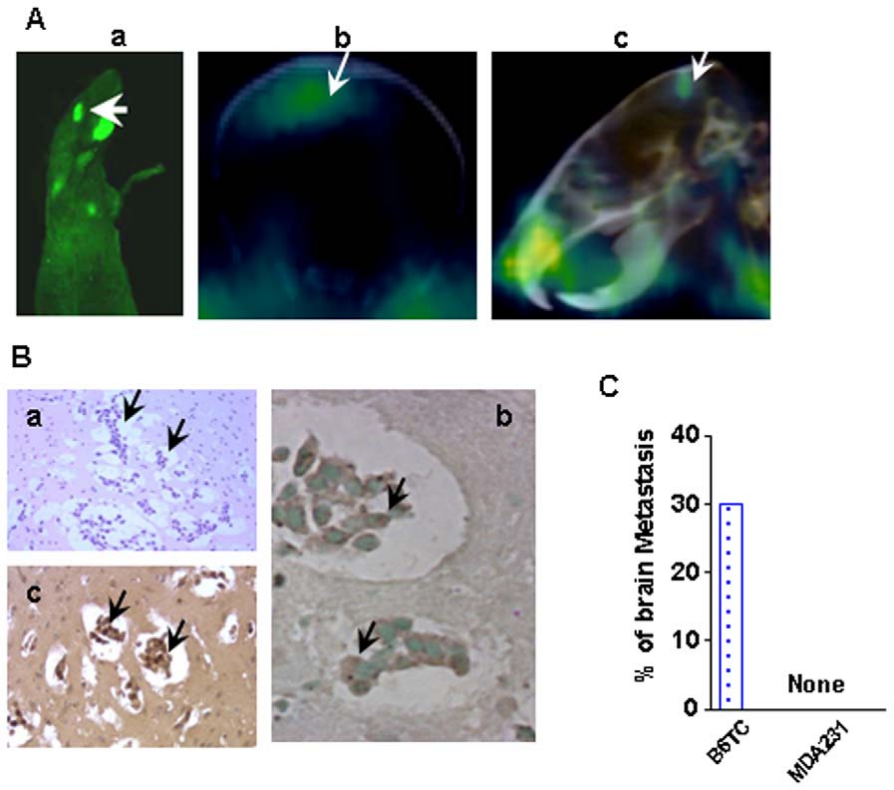
Brain metastasis induced by intracardiacally inoculated B6TC cells. (A) Representative pictures of brain metastases of the B6TC cell-inoculated mice as detected by GFP whole mouse imaging (Panel a) and microPET/CT imaging (Panels b and c). (B) Brain metastatic B6TC cells were detected with H&E staining (Panel a), immunohistochemical staining for GFP or TP53 as shown in Panels b and c, respectively. (C) Percentage of brain metastases in B6TC and MDA-MB-231 groups as detected by the GFP imaging.

During the whole mouse imaging for GFP-expressing metastatic tumors at Week 4 after intracardiac inoculation of the cancer cells, we noticed tumors in the brain area of B6TC mice (Fig. 6Aa). PET/CT imaging confirmed that the tumors are in the brain, not in calvarial bone (Fig. 6Ab and c). After the termination of the experiment, the brain was removed, bisected, and 10 sagittal sections were prepared at 300-µm intervals through one hemisphere. The sections were processed for either H&E staining or for immunohistochemistry staining for GFP or human TP53. The presence of brain metastases was confirmed by the presence of tumor cells in the H&E stained brain sections (Fig. 6Ba), which were confirmed to be human cancer cells since they expressed GFP (Fig. 6Bb) and human TP53 (Fig. 6Bc). Whole mouse GFP and PET/CT imaging revealed that thirty percent of the B6TC mice had brain metastases whereas none of the MDA-MB-231 mice showed brain metastasis at the end of the fourth week (Fig. 6C). On the other hand, we found that ZR-75-1 cells can induce brain metastasis when inoculated through intracardiac injection in the presence of exogenous estrogen supplementation (Fig. S3) suggesting that the brain metastatic property of B6TC cell may be derived from ZR-75-1 cell.

### B6TC cell exhibits more prominent tumor stem cell features than its parental cells

**Figure 7:**
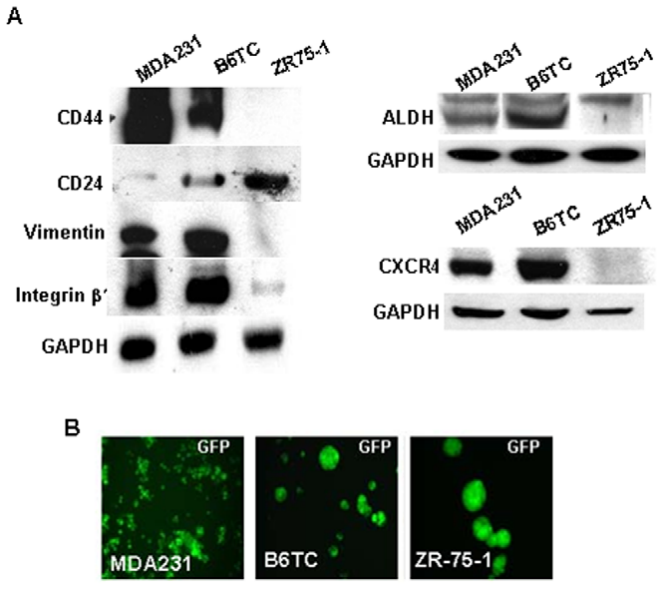
Comparison of cancer stem cell features of B6TC cell with its parental cells. (A) Western blot analysis for stem cell markers of CD44, CD24, Vimentin, Integrin β1, ALDH, and CXCR4 in the cell extracts of the ZR-75-1, B6TC and MDA-MB-231 cells. GAPDH levels were measured for equal loading. (B) Formation of mammospheres by the three cell lines in suspension culture with GFP imaging.

Cancer cells with high metastatic potential are believed to have a higher population of cancer stem cells with epithelial to mesenchymal transition properties. Being highly metastatic, B6TC cells were found to express intermediate levels of CD44 and CD24 in comparison to its parental MDA-MB-231 and ZR-75-1 cells (Fig. 7A). But, like MDA-MB-231 cell, it expressed high levels of other stem cell markers including Vimentin, Integrin β1, ALDH, and CXCR4 (Fig. 7A). On the other hand, similar to ZR-75-1 cells, B6TC cells were capable of forming mammospheres when the cells were suspended in a dedifferentiation medium exhibiting cancer stem cell properties, whereas MDA-MB-231 cells were incapable of forming mammospheres which are characteristic of these cells (Fig. 7B).

## Discussion

It has been indicated previously that spontaneous cell fusion between tumor cells or between tumor cells and bone marrow derived stem cells might play an important role in the acquisition of malignant phenotypes including aneuploidy, drug resistance, and cancer stem cell features [9]–[11]. For example, fusion of two cell lines, which were resistant to two different drugs, produced a new cell line that was not only resistant to the two drugs, but also resistant to an additional drug [12]. A recent study from Yibin Kang's group showed spontaneous cell fusion in vitro or in vivo between two sublines of the MDA-MB-231 breast cancer cell line with different metastasis organotropisms resulting in the formation of a stable new cell line with dual metastasis organotropisms [4]. In this study, we have generated an estrogen independent highly malignant B6TC hybrid cell line, spontaneously formed between an ERα positive and ERα negative cell, and isolated from a bone marrow metastatic site. Since B6TC cells are much more malignant than its prarental cells with a number of stem cell characteristics, we speculate that it might be formed by the fusion of at least one cell with stem cell features.

Our study was initiated to investigate whether the presence of primary tumors formed by highly metastatic MDA-MB-231 or MDA-MB-435 cells can drive less metastatic ZR-75-1 cell homing to bone. In the process of characterizing bone metastatic ZR-75-1 cells, we identified the hybrid cell line, B6TC. Whether a specific metastasis-permissive niche in the bone established by the primary tumors might be involved in the generation of this hybrid cell line would require further investigation. The B6TC cells originated from B6 cells, which were generated in the bone marrow, and it was composed of a mixed population of ER high and low cells with marked differences in their proliferative potential and malignancy. Among them, the low ER expressing B6TC cells are highly metastatic. B6TC cell contains the identical p53^R280K^ mutation as the MDA-MB-231 cell and in large part resembles MDA-MB-231 cell with respect to its growth property and response to estrogen. However, its brain metastatic property and ability to form mammospheres appears to resemble ZR-75-1 cell rather than MDA-MB-231 cell. Gene expression profiling indicates that B6TC cell is less different from MDA-MB-231 cell than from ZR-75-1 cell. We are currently in the process of identifying genes that contribute to the bone and brain metastatic property of B6TC cell.

The precise cellular and molecular mechanisms that lead to metastases of a tumor to a pre-determined location are not known. To specifically identify genes that mediate metastasis, animal models have been used to select in vivo for highly metastatic and organ specific derivatives of human cancer cell lines [13]. In this study, the B6TC cells are highly metastatic to the bone as well as to the brain. Bone is the most common site for cancer metastases. Breast cancer metastasizes to bone in more than 80% of patients with advanced disease [14]. Existing evidences suggest that enhanced formation and activation of osteoclasts induced by colonization of cancer cells are critical to the establishment of bone metastases. B6TC cells consistently caused osteolytic bone metastases in nude mice after intracardiac inoculation. This model provides opportunity to study pathogenesis and molecular mechanism of tumor cell-bone interactions in breast cancer metastases. Our results demonstrate B6TC tumor causes increased bone resorption, not counterbalanced by bone formation. Future investigation is needed to define the role of different tumor producing osteoclastogenic factors, which stimulates the differentiation and activation of the osteoclasts, leading to the progression of bone destruction in the steps of bone metastatic cascade of B6TC cells. This model can be used in search of effective treatment for osteoclastic bone metastasis.

The RNA microarray study for identifying genes that are unique and overexpressed in B6TC cells compared to the parental cell lines MDA-MB-231 and ZR-75-1 and characterization of those genes with respect to their biological function will certainly have great impact on our current understanding of metastasis. Brain metastases are the most feared complication in breast cancer. Nearly 20% of patients with advanced breast cancer are eventually diagnosed with brain lesions, making breast tumors the main source of metastatic brain disease in women [15]–[17]. Because of the availability of many advanced cancer therapeutics, the life span of breast cancer patients has been increased. However, presently available treatment regimens are not effective in treating breast cancer brain metastases. Limited rodent model systems have been reported for brain metastasis in breast carcinoma [18], [19]. Therefore, there is a need of efficient model systems that can be utilized for our understanding of different factors from both the host and the tumor contributing to brain metastasis. Overexpression of the genes related to brain colonization in our B6TC model may reveal novel genes contributing to the brain metastasis by comparing the signal pathways involved. The brain tissues are well-protected by blood brain barrier [17]. Intravasation of such complex barrier occurs at a very low rate compared to the intravasation in a lung capillary. Also, the unique microenvironment present in the brain is relatively poorly understood, but seems to be different from other organs and may influence brain metastases [17], [20], [21]. There are reports that CXCR4 may act as a signature gene in contributing to the invasive and metastatic behavior of the breast cancer cells to the brain [22]. Our gene profiling data also implicates CXCR4 in brain metastasis as it is upregulated in B6TC in comparison to its parental cells. The brain permeability is very important for drug development [17], [21]. The understanding of brain metastasis in our B6TC model will provide insights into the development of potential novel therapies for breast cancer-induced brain metastasis, especially for the search of efficacious small inhibitors that can cross the blood brain barrier.

Many solid tumor types, including breast cancer, exhibit a functional hierarchy of cancer cells of which only a small subpopulation of stem-like cells can give rise to the differentiated cells that comprise the bulk tumor [23]–[28]. The cancer stem cell hypothesis is very important concept in cancer biology and also for the detection, prognosis and prevention of cancer. The development of cancer therapeutics based on tumor regression may have produced agents which kill differentiated tumor cells while sparing the small cancer stem cell population [29]. Therefore development of successful cancer therapeutic regimen requires targeting this cancer stem cell population. Such cells represent a subpopulation of cancer cells that, by one mechanism or another, have the capacity to act as tumor-propagating cells [30], [31]. In our study, we show that B6TC cells express a high level of CD44+ and a low level of CD24−, a phenotype similar to the cancer stem cells. One candidate stem cell marker with conserved stem and progenitor cell functions is aldehyde dehydrogenase 1 (ALDH1), a detoxifying enzyme responsible for the oxidation of intracellular aldehydes [32]–[35]. It has been shown that murine and human hematopoietic and neural stem and progenitor cells have a high ALDH activity [36]–[39]. The B6TC cell populations show high expression of ALDH. Since it has been shown that cells with high ALDH activity contain the tumorigenic cell fraction, are able to self-renew, and recapitulate the heterogeneity of the parental tumor [40], B6TC cells likely have a population of cancer stem cells. The formation of mammospheres by B6TC cells confirms that it has breast cancer stem cells as mammospheres generated from normal mammary epithelium are enriched in stem/progenitor cells [41]. Therefore, it will be of great interest to determine whether these CD44+/CD24−/ALDH+ cells are directly targeted by alternative therapies, such as novel small molecule inhibitors. Based on our results, using B6TC model to target CD44+/CD24−/ALDH+ cells for drug discovery offers a highly promising and reproducible means to identify therapies that prevent self-renewal of cancer stem cells in a tumor microenvironment.

In summary, our study shows that tumors formed by aggressive ER-negative breast cancer cells can enhance metastatic potential of less aggressive ER-positive breast cancer cell. Cell fusion may be a unique mechanism of tumor heterogeneity. The estrogen-independent highly malignant B6TC hybrid cell line with stem cell-like properties, spontaneously formed between an ERα positive and ERα negative cell and isolated from a bone marrow metastatic site, will be useful for the investigation of the molecular mechanism of brain metastasis and for the therapeutic targeting of breast cancer cells resistant to anti-estrogens.

## Materials and Methods

### Ethics statement

All animal experiments were conducted following appropriate guidelines. They were approved by the ethics committee/institutional review board, ‘Institutional Animal Care and Use Committee’ (IACUC approval ID 99142X3411A) and monitored by the Department of Laboratory Animal Resources (DLAR) at the University of Texas Health Science Center at San Antonio.

### Cell lines and culture

MDA-MB-435-F-L/GFP cells, which are highly invasive and metastatic variants of human carcinoma MDA-MB-435 cells, were isolated in our laboratory [42]. The human breast cancer cell lines ZR-75-1 was originally obtained from the American Type Culture Collection (Manassas, VA) and stably transfected with the enhanced green fluorescent protein (GFP) for the detection of metastases in vivo with green fluorescence imaging. We used a clone of GFP-transfected MDA-MB-231 cells named clone 10 from Dr. Julie Anne Sterling at Vanderbilt University, which was resistant to G418 antibiotic. This clone, like its parent line, induced lung metastases when growing orthotopically and both lung and bone metastases when inoculated intracardiacally in our preliminary tests, and is hereafter referred as MDA-MB-231 cell. ZR-75-1/GFP cell was also stably transfected with a puromycin-resistant gene expression vector and puromycin-resistant cells were pooled and used in the experiments. These cell lines were cultured in McCoy's 5A medium supplemented with pyruvate, vitamins, amino acids, antibiotics, and 10% fetal bovine serum, as previously described [43], Working cultures were maintained at 37°C in a humidified incubator with 5% CO_2_.

### Antibodies

Antibody to the Estrogen Receptor alpha was from Labvision Corporation (Fremont, CA), antibody to Vimentin was from Sigma Aldrich (St. Louis, MO), antibody to CXCR4 was from Abcam (Cambridge, MA), and antibodies to ALDH, CD44, CD24 and Integrin β_1_ were from Santa Cruz Biotechnology (Santa Cruz, CA).

### Western blot analysis

Cells were grown to confluence in 60-mm tissue culture dishes and lysed in a buffer solution [50 mmol/L Tris-HCl (pH 7.4), 150 mmol/L NaCl, 0.5% NP40, and a protease inhibitor cocktail from Pierce, Rockford, IL]. Equal amounts of protein were separated on 10% or 7.5% SDS-PAGE gels and blotted onto nitrocellulose membranes. The blotted membranes were incubated with respective primary antibodies; mouse anti–glyceraldehyde-3-phosphate dehydrogenase (GAPDH; 1∶10,000; Ambion, Inc., Austin, TX) was used as a loading control, horseradish peroxidase–conjugated goat anti-rabbit (1∶5,000; Santa Cruz Biotechnology), and horseradish peroxidase–conjugated goat anti-mouse (1∶5,000; Santa Cruz Biotechnology) antibodies were used as secondary antibodies.

### TGF-β sensitivity assay

For the measurement of the transcriptional activity of TGF-ß with a TGFß responsive promoter-luciferase construct, pSBE4-Luc, cells were seeded at 2.0×10^4^ per well in 12-well plates. When cells were ∼80% confluent, they were cotransfected with 1.0 µg of pSBE4-Luc and 0.5 µg of a ß-galactosidase expression plasmid using 4.5 µL of Lipofectamine 2000 (Invitrogen, Carlsbad, CA) in serum-free medium following the manufacturer's protocol. After 4 h, the medium was replaced with the serum-containing medium and TGFß3 was added at 5.0 ng/mL. The cells were lysed after overnight incubation in buffer (100 mmol/L K_2_HPO_4_, 1 mmol/L DTT, and 1% Triton X-100) and the luciferase in the cell lysate was measured as previously described [8]. Luciferase activity was activity normalized for transfection efficiency by β-galactosidase activity.

### Cell proliferation assay

Cells were plated in 96-well plates at 1,600 cells/well. Relative cell number in each well was determined every 24 h by MTT assay as described previously [44].

### Soft-Agarose assay

To determine the ability of the cell to grow anchorage-independently in a semisolid medium, soft agarose assays were performed as described previously [44]. Briefly, 6×10^3^ cells were suspended in 1 ml of 0.4% low melting point agarose (Life Technologies, Carlsbad, CA) dissolved in the regular culture medium and plated on the top of a 1 ml underlayer of 0.8% agarose in the same medium in 6-well culture plates. After 3–4 weeks of incubation in the humidified incubator with 5% CO_2_ at 37°C, the cell colonies were visualized by staining with 1 ml of p-iodonitrotetrazolium violet staining (Sigma). Cell colonies were counted.

### Tumorigenicity and in vivo lung metastatic study

Four- to five-week-old female athymic nude mice (Harlan Sprague Dawley, Inc., Indianapolis, IN) were used for *in vivo* animal experiments. The animals were housed under specific pathogen-free conditions. All animal protocols were approved and monitored by the institutional animal care and use committee.

EGFP-expressing MDA-MB-435-F-L, MDA-MB-231, ZR-75-1/puro cells and its variant lines B4, B6, and B6TC cells were separately inoculated (2×10^6^ cells) into the both inguinal mammary fat pad of 5-week-old female athymic nude mice. The tumor sizes were measured with a caliper in two dimensions. Tumor volumes were calculated with the equation V =  (L×W^2^) x 0.5, where L is length and W is width of a tumor. Animals were sacrificed 4 or 6 weeks after tumor cell inoculation. The green metastatic cancer cell colonies of different sizes were visually observed, counted and measured in the whole lungs using NIKON fluorescence microscope (TE-200) with a 20X objective (200X magnification).

### Establishment of cell lines from xenograft tumors

The orthotopic tumors formed by B6 cells in the presence or absence of estrogen supplementation were excised from mice inside a tissue culture hood and cut into 2–3 mm pieces in a 100 mm sterile Petri dish containing a serum-free (SM) medium. The tumor tissues were transferred to a 50-ml tube containing 10 ml SM medium and 100 µl (equivalent to 2.8 units) of Liberase Blendzyme (Roche, Indianapolis, IN). The tube was incubated in a CO_2_ incubator at 37°C for 30–45 minutes, vortexed at 10 min. interval, filtered through a 70 micron membrane filter (Fisher) and centrifuged at 500 g for 5 minutes at 4°C. The supernatant was discarded and the cell pellet was resuspended and cultured in McCoy's 5A medium containing 10% fetal bovine serum. After 2–3 days, medium was changed to the medium containing G418 to kill the mouse cells and select for the cancer cells. The selected B6TC and B6TE cells were cryopreserved in liquid N_2_ for future use.

### Experimental in vivo bone and lung metastasis assay

An intracardiac injection model for experimental bone metastasis was used for this study, as previously described. Briefly, MDA-MB-231, ZR-75-1, B6, B6TC cells were harvested from subconfluent exponentially growing cultures. The cells were injected into the left cardiac ventricle of anesthetized female nude mice (5 weeks old) with a 27-gauge needle attached to a 1-ml syringe using a micromanipulator. Each mouse was injected with 10^5^ cells in 0.1 ml of phosphate-buffered saline, and successful injections were indicated by the pumping of red blood into the syringe. Development of bone metastasis induced by EGFP-expressing cells was monitored at regular intervals by whole-animal green fluorescence imaging to detect EGFP-expressing tumor cells growing in the legs, arms, spine, and mandible bones, using a Nikon SMZ1500 (Nikon, Tokyo, Japan) fluorescence stereoscope attached to a CoolSNAP CCD camera (Photometrics, Tucson, AZ). Radiographs taken with Faxitron (Lincolnshire, IL) were used for the detection of any bone lesion in mice injected with cancer cells. At the termination of experiment after about 4 weeks, whole lungs were excised, and EGFP-expressing metastatic cancer cell colonies were visually observed and counted under an inverted fluorescence microscope. Bone tissues were fixed in 10% neutral-buffered formalin (Fisher Scientific, Houston, TX) for 24 hours at room temperature, decalcified in 10% EDTA, and embedded in paraffin. Sections were stained with hematoxylin, eosin, orange G, and phloxine, and the presence of metastatic tumors and bone osteolysis in the femora and tibiae was examined under a microscope.

### Radiolabeling and quality control

Bevacizumab (Avastin, Genetech) are radiolabeled with positron emitter ^64^Cu (^64^CuCl_2_ in 0.1 M HCl; radionuclide purity >99%, University of Wisconsin). For radiolabeling, Wipke and Wang's method was applied [45], [46]. Briefly, Bevacizumab was reacted with a 100∶1 molar ratio of DOTA-NHS-ester (1,4,7,10-tetraazacyclododecane-N,N′,N″,N′″-tetraacetic acid mono-N-hydroxysuccinimide ester, M.W =  990 mg/mmol): antibody in 0.1 M Na_2_HPO_4_ buffer of pH 7.5 at 4°C for 12–16 h. After conjugation, the reaction mixture was centrifuged repeatedly through a YM-30 centricon with 0.1 M ammonium citrate buffer of pH 6.5 in order to remove unconjugated small molecules. The concentration of purified antibody-conjugate was determined by a UV spectrophotometer measuring the absorbance at 280 nm. Typically, 1 mg of DOTA-conjugated antibody and 5 mCi of ^64^Cu were incubated in 0.1 M ammonium citrate, pH 6.5, at 43°C for 45 minutes. Labeled antibody was separated by a size-exclusion column (Bio-Spin6, BIO-RAD Laboratories, Hercules, CA). Radiochemical purity of antibody was determined by integrating areas on the Fast Protein Liquid Chromatography (FPLC) with a size-exclusion column and measuring the percentage of radioactivity associated with the 150 kDa protein peak. The stability of the ^64^Cu radiolabeled molecules was determined by bovine serum challenge at 24 hours.

### Methods for MicroCT and ^64^Cu imaging

The distribution of ^64^Cu-bevacizumab was investigated in B6TC bone metastasis nude mice model. Each mouse was injected intravenously via a lateral tail vein with ^64^Cu-bevacizumab at the dose of 200 µCi/40 µg mAb in 200 µl of saline. Mice were scanned at 1, 4, 24, and 48 h after injection for microCT and ^64^Cu imaging. All the mice were anesthetized with 2–3% isoflurane in 100% oxygen during injection and scanning. With the mice kept prone on the bed of the Gamma Medica-Ideas FLEX preclinical system, static PET images were acquired for 10 minutes at 1 and 4 h post-injection, 15 minutes at 24 h post-injection, and 20 minutes at 48 h post-injection to monitor ^64^Cu-bevacizumab distribution. The whole mouse was in the field of view. MicroCT images (75 kVp, 0.25 mA, 360° rotation, 256 projections) were acquired before each PET imaging session using fly mode to acquire quality image and decrease radiation dose to the mice. The images were reconstructed by using the software offered from Gamma Medica-Ideas Inc and were fused by Amira based software VIVID.

### Mammosphere culture

Cells were cultured as mammospheres in anchorage independent conditions as described earlier [47]. Briefly, Single cells were plated in ultralow attachment 24 well plates (Corning) at a density of 10,000 viable cells/ml. Cells were grown in a serum-free DMEM-F12 medium (Gibco, Carlsbad, CA) supplemented with B27 (Invitrogen, Carlsbad, CA 1∶50), 20 ng/ml EGF and 20 ng/mL bFGF (BD Biosciences, San Jose, CA), and 4 µg/ml heparin (Sigma). Culture medium contained 1% methyl cellulose to prevent cell aggregation. Colonies were monitored microscopically daily to ensure that they were derived from single cells.

### Microarray processing

Total RNA was extracted from near confluent B6TC, MDA-MB-231/GFP/Neo and ZR-75-1/GFP/puro cells. The RNA was purified using a RiboPure Kit from Ambion following the manufacturer's suggested protocol. RNA quality and quantity was ensured using the Bioanalyzer (Agilent, Inc., Santa Clara, CA) and NanoDrop (Thermo Scientific.,Waltham, MA) respectively. Per RNA labeling, 5 micrograms of total RNA was used in conjunction with the Affymetrix recommended protocol, One-Cycle Target Labeling and Control Reagents.

The hybridization cocktail containing the fragmented and labeled cDNAs was hybridized to The Affymetrix GeneChip® Human Genome U133 Plus 2.0 Array. The chips were washed and stained by the Affymetrix Fluidics Station using the standard format and protocols as described by Affymetrix. The probe arrays were stained with streptavidin phycoerythrin solution (Molecular Probes, Carlsbad, CA) and enhanced by using an antibody solution containing 0.5 mg/mL of biotinylated anti-streptavidin (Vector Laboratories, Burlingame, CA). An Affymetrix Gene Chip Scanner 3000 was used to scan the probe arrays. Gene expression intensities were calculated using the Gene Chip Operating software 1.2 (GCOS 1.2, Affymetrix). Each cell line was done in triplicate labeling and hybridization.

Cell files were used for the GC-corrected robust multichip average (GC-RMA) to adjust for background and non-specific binding and to normalize expression values across arrays. Non-specific filtering was used to remove probe-sets that showed very low expression levels across a majority of the arrays and little differential variation across arrays. An empirical Bayes modified robust t statistics was used to compare differences in expression levels for each gene between cell lines, and we used the False Discovery Rate method to adjust for multiple testing effects. Hierarchical clustering was used to visualize patterns of significantly down- and up-regulated genes across arrays. Expression values are standardized prior to clustering. The joint solution is displayed in a false color image called a heatmap that displays gene clusters as rows and array clusters as columns. The red to blue color scheme reveals the pattern of down- and up-regulated genes that separate the cell lines. All analyses were conducted using procedures in the R/Bioconductor suite [48]. All microarray data is MIAME compliant and the raw data has been deposited in the MIAME compliant database GEO (Accession number GSE27515).

## Supporting Information

Figure S1 A. Gene expression profile of B6TC, MDA-MB-231 and ZR-75-1 cells represented by heatmap. B. The results from triplicate microarray chips revealed that B6TC cell has a different gene expression profile from those of its two parent cell lines.
(TIF)

Figure S2 ***TP53*** **sequence comparison among the B6TC, MDA-MB-231 and ZR-75-1 cells.** Briefly, total RNA was extracted from near confluent B6TC, MDA-MB-231, and ZR-75-1 cells. The RNA was purified using a RiboPure Kit from Ambion following the manufacturer's suggested protocol. Total RNA sample were reverse transcribed with random hexamers. The cDNAs were amplified in PCR with *TP53* sense and antisense. The PCR product was purified using the PCR cleaning kit from Qiagen (Hilden, Germany) and sequenced. The sequence shown in figure is antisense sequence where TCT codes for Arginine (found in wild type *TP53* in ZR-75-1 cell) and TTT codes for Lysine (found in mutated *TP53* in MDA-MB-231 and B6TC cells).
(TIF)

Figure S3 **Representative picture of brain metastasis induced by ZR-75-1 cells as detected by GFP imaging.** In an intracardiac injection model, 0.1×10^6^ ZR-75-1 cells were injected into the left cardiac ventricle of female nude mice supplemented with a 17β-estradiol (E2) pellet. Two out of four mice developed metastases to the brain as indicated by the presence of green fluorescence metastatic lesions in the brain.
(TIF)
