# Supplementary figures and images for: Isolation and Characterization of a Metastatic Hybrid Cell Line Generated by ER Negative and ER Positive Breast Cancer Cells in Mouse Bone Marrow
Source: PLoS One. 2011 Jun 1;6(6):e20473. doi: 10.1371/journal.pone.0020473 (PMC3106006; doi:10.1371/journal.pone.0020473)

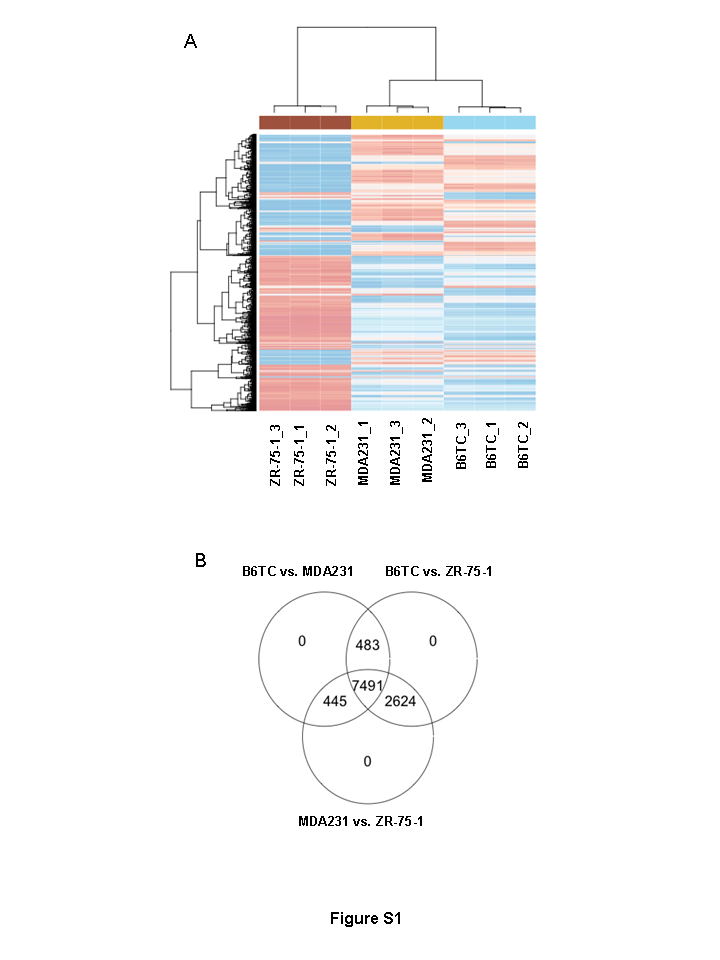

Supplement: Figure S1 — A. Gene expression profile of B6TC, MDA-MB-231 and ZR-75-1 cells represented by heatmap. B. The results from triplicate microarray chips revealed that B6TC cell has a different gene expression profile from those of its two parent cell lines. (TIF) [file pone.0020473.s001.tif]

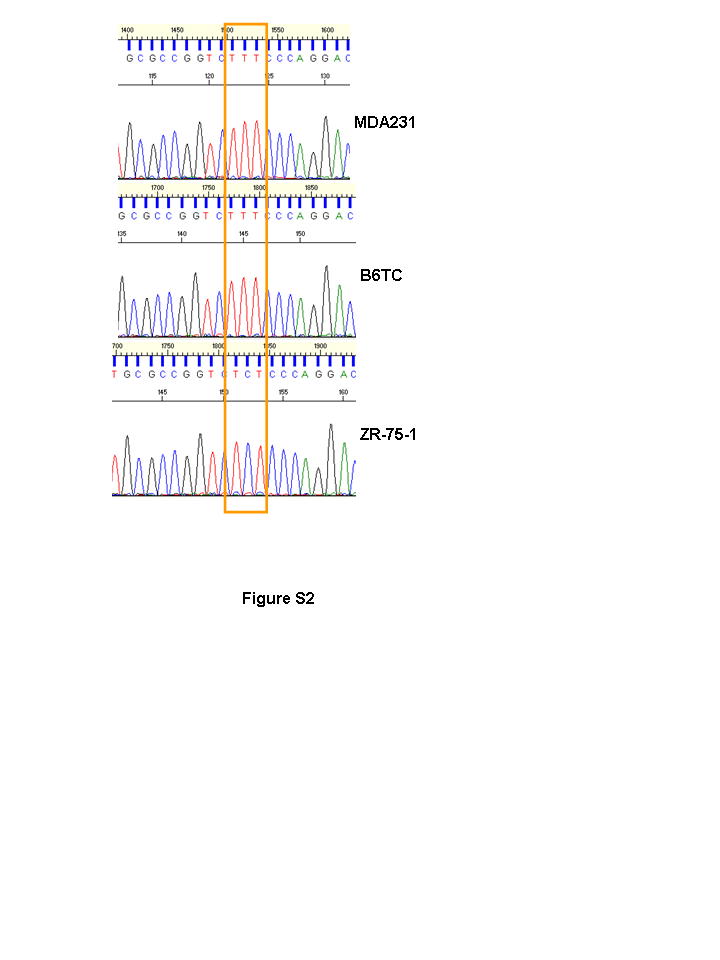

Supplement: Figure S2 — TP53 sequence comparison among the B6TC, MDA-MB-231 and ZR-75-1 cells. Briefly, total RNA was extracted from near confluent B6TC, MDA-MB-231, and ZR-75-1 cells. The RNA was purified using a RiboPure Kit from Ambion following the manufacturer's suggested protocol. Total RNA sample were reverse transcribed with random hexamers. The cDNAs were amplified in PCR with TP53 sense and antisense. The PCR product was purified using the PCR cleaning kit from Qiagen (Hilden, Germany) and sequenced. The sequence shown in figure is antisense sequence where TCT codes for Arginine (found in wild type TP53 in ZR-75-1 cell) and TTT codes for Lysine (found in mutated TP53 in MDA-MB-231 and B6TC cells). (TIF) [file pone.0020473.s002.tif]

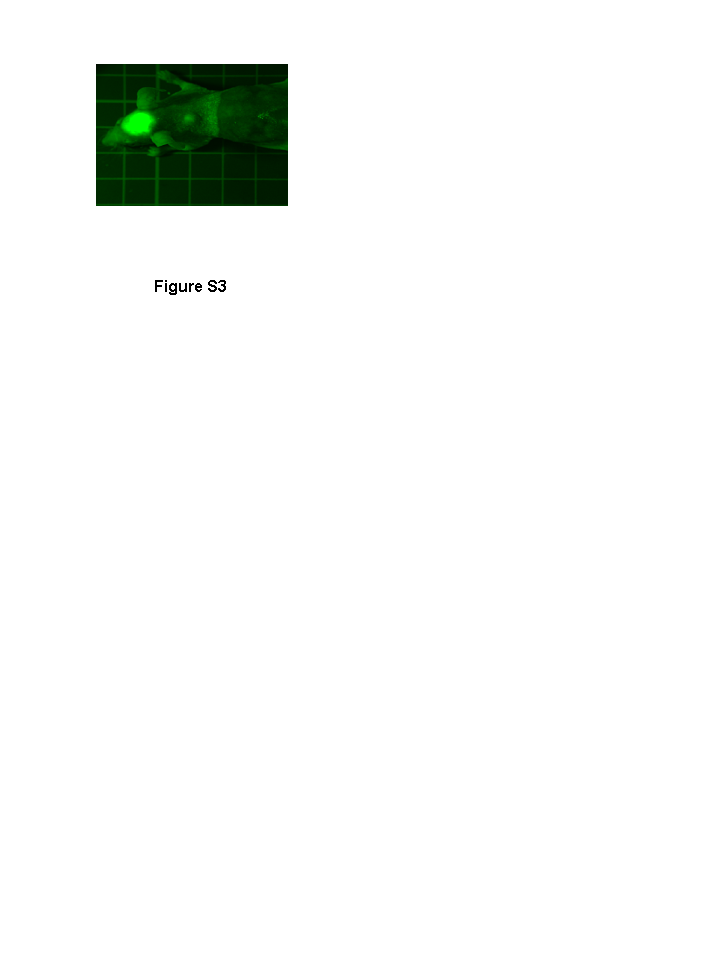

Supplement: Figure S3 — Representative picture of brain metastasis induced by ZR-75-1 cells as detected by GFP imaging. In an intracardiac injection model, 0.1×10^6^ ZR-75-1 cells were injected into the left cardiac ventricle of female nude mice supplemented with a 17β-estradiol (E2) pellet. Two out of four mice developed metastases to the brain as indicated by the presence of green fluorescence metastatic lesions in the brain. (TIF) [file pone.0020473.s003.tif]
